# Diffusion tensor imaging for characterizing tumor microstructure and improving diagnostic performance on breast MRI: a prospective observational study

**DOI:** 10.1186/s13058-019-1183-3

**Published:** 2019-09-04

**Authors:** Jing Luo, Daniel S. Hippe, Habib Rahbar, Sana Parsian, Mara H. Rendi, Savannah C. Partridge

**Affiliations:** 1 Department of Radiology, University of Washington School of Medicine, 825 Eastlake Avenue East, Seattle, WA 98109 USA; 2 Department of Pathology, University of Washington School of Medicine, 1959 NE Pacific St. Box 356100, Seattle, WA 98195 USA; 3 Department of Radiology, Seattle Cancer Care Alliance, 1144 Eastlake Ave E, LG2-200, PO Box 19023, Seattle, WA 98109 USA

**Keywords:** Diffusion tensor imaging (DTI), Dynamic contrast-enhanced (DCE) MRI, Breast MRI, Apparent diffusion coefficient (ADC), Fractional anisotropy (FA), Suspicious lesions, False positives, Diagnosis

## Abstract

**Background:**

Diffusion-weighted imaging (DWI) can increase breast MRI diagnostic specificity due to the tendency of malignancies to restrict diffusion. Diffusion tensor imaging (DTI) provides further information over conventional DWI regarding diffusion directionality and anisotropy. Our study evaluates DTI features of suspicious breast lesions detected on MRI to determine the added diagnostic value of DTI for breast imaging*.*

**Methods:**

With IRB approval, we prospectively enrolled patients over a 3-year period who had suspicious (BI-RADS category 4 or 5) MRI-detected breast lesions with histopathological results. Patients underwent multiparametric 3 T MRI with dynamic contrast-enhanced (DCE) and DTI sequences. Clinical factors (age, menopausal status, breast density, clinical indication, background parenchymal enhancement) and DCE-MRI lesion parameters (size, type, presence of washout, BI-RADS category) were recorded prospectively by interpreting radiologists. DTI parameters (apparent diffusion coefficient [ADC], fractional anisotropy [FA], axial diffusivity [*λ*_1_], radial diffusivity [(*λ*_2_ + *λ*_3_)/2], and empirical difference [*λ*_1_ − *λ*_3_]) were measured retrospectively. Generalized estimating equations (GEE) and least absolute shrinkage and selection operator (LASSO) methods were used for univariate and multivariate logistic regression, respectively. Diagnostic performance was internally validated using the area under the curve (AUC) with bootstrap adjustment.

**Results:**

The study included 238 suspicious breast lesions (95 malignant, 143 benign) in 194 women. In univariate analysis, lower ADC, axial diffusivity, and radial diffusivity were associated with malignancy (OR = 0.37–0.42 per 1-SD increase, *p* < 0.001 for each), as was higher FA (OR = 1.45, *p* = 0.007). In multivariate analysis, LASSO selected only ADC (OR = 0.41) as a predictor for a DTI-only model, while both ADC (OR = 0.41) and FA (OR = 0.88) were selected for a model combining clinical and imaging parameters. Post-hoc analysis revealed varying association of FA with malignancy depending on the lesion type. The combined model (AUC = 0.81) had a significantly better performance than Clinical/DCE-MRI-only (AUC = 0.76, *p* < 0.001) and DTI-only (AUC = 0.75, *p* = 0.002) models.

**Conclusions:**

DTI significantly improves diagnostic performance in multivariate modeling. ADC is the most important diffusion parameter for distinguishing benign and malignant breast lesions, while anisotropy measures may help further characterize tumor microstructure and microenvironment.

## Background

Dynamic contrast-enhanced (DCE) breast MRI is established to be the most sensitive tool for the detection of breast cancer [1–5]. As a result, its utility in both screening and diagnostic setting has increased rapidly over the past two decades. Although breast MRI specificity and positive predictive value have improved since its inception, DCE MRI continues to result in many false positives and unnecessary biopsies [2, 6, 7]. In fact, recent studies have demonstrated that as few as one in five biopsy recommendations based on DCE MRI yield malignancy [8–10]. These unnecessary biopsies can result in increased health care costs, patient anxiety, and delays in breast cancer treatment. Accordingly, identification of MRI methods that complement DCE techniques and improve breast MRI specificity without lowering its sensitivity is an important area of active research.

Diffusion-weighted imaging (DWI) has emerged as an adjunct to DCE-MRI that can improve the detection and characterization of breast cancer [11–13]. DWI interrogates the in vivo mobility of water molecules, which in turn may provide information on microstructural characteristics of tissue, including cell density and presence of macromolecules and cell membranes. Numerous prior studies have shown that breast cancers feature impeded diffusion and appear as the areas of hyperintensity on DWI with correspondingly low apparent diffusion coefficient (ADC) compared to normal fibroglandular tissue [14]. The best explored application of DWI for breast cancer is decreasing the false-positive rate and increasing the diagnostic specificity when used in addition to conventional DCE-MRI [15, 16]. Although DWI is prone to susceptibility and field inhomogeneity artifacts, diffusion sequences can be acquired quickly and can help to identify lesions warranting biopsy [14].

Diffusion tensor imaging (DTI) is an extension of conventional DWI that interrogates water motion in six or more directions to characterize diffusion directionality (anisotropy) in addition to ADC [17]. Water diffusion within biological tissue is often anisotropic due to directionally dependent restriction imposed by microstructural architecture. It is hypothesized that normal mammary ducts allow water to diffuse more freely in a direction parallel to the walls of the ducts whereas proliferating neoplastic cells reduce diffusion anisotropy by blocking ducts [18, 19]. Although there is a general consensus that malignant lesions demonstrate reduced diffusion on DWI in comparison with most benign and normal fibroglandular tissues, there are conflicting results regarding the added diagnostic utility of DTI parameters such as fractional anisotropy (FA), a measure of diffusion directionality in which higher FA indicates more anisotropic diffusion oriented along a single direction and lower FA indicates more equal diffusion in all directions [18, 20–24]. Some studies have reported lower FA in benign lesions compared with malignant lesions, attributed to the differences in microscopic composition and organization [20, 23–25], while others found no such differences in FA [18, 21, 22].

The purpose of our study was to evaluate the DTI features of suspicious breast lesions detected on 3 T MRI and to determine whether DTI can statistically improve diagnostic performance over conventional assessment.

## Methods

### Study population

This prospective study was approved by our Institutional Review Board and was compliant with the Health Insurance Portability and Accountability Act (HIPAA). All patients provided informed consent allowing us to review the MRI images, medical records, and pathology results. Enrolled patients were 18 years or older and underwent 3 T breast MRI, including DCE and DTI sequences, from October 2010 to December 2013. DTI sequences were appended to the standard clinical MRI examination; therefore, enrolled patients did not undergo additional MRI examinations. Patients with MRI-detected lesions characterized as Breast Imaging Reporting and Data System (BI-RADS) category 4 or 5 who underwent core needle biopsy (CNB) and/or surgical excision were eligible for the study. Clinical indications for breast MRI included high-risk screening, extent of disease evaluation, and problem solving. In subjects with known existing breast cancer, the eligible lesion must have been distinct from the previously biopsy-proven cancer. Subjects receiving neoadjuvant chemotherapy less than 6 months prior to MRI were excluded. Patients unable or unwilling to provide informed consent or undergo the entire MRI examination were also excluded.

### MRI acquisition

Breast MRIs were performed using a 3 T Philips Achieva Tx MRI scanner (Philips Healthcare, Best, The Netherlands) with a dedicated 16-channel bilateral breast coil (Mammo-Trak, Philips Healthcare, Best, The Netherlands). MRI sequences were obtained in the axial orientation, and each MRI exam included DWI, T2-weighted fast spin-echo, T1-weighted non-fat-suppressed, and T1-weighted fat-suppressed DCE-MRI sequences with one precontrast and three post-contrast acquisitions. DCE-MRI was acquired with T1-weighted fat-suppressed 3D fast gradient echo (eTHRIVE) sequences with parallel imaging technique (sensitivity encoding; SENSE). The following imaging parameters were utilized: repetition time (TR)/echo time (TE), 5.9/3 ms; flip angle, 10°; matrix size, 440 × 660; field of view (FOV), 22 × 33 cm; in-plane voxel size, 0.5 mm; and slice thickness, 1.3 mm. Post-contrast sequences were acquired with *k*-space centered at 120, 300, and 480 s after contrast injection. The contrast agent administered was 0.1 mmol/kg body weight gadoteridol (ProHance, Bracco Diagnostics, Milan, Italy). The DCE scan time was 2 min and 57 s per acquisition.

In order to minimize disruption of the clinical portion of breast MRI examinations, diffusion tensor imaging was performed immediately following DCE imaging using a 2D diffusion-weighted single-shot spin-echo-prepared echo-planar imaging (EPI) sequence with parallel imaging and fat suppression (spectral attenuated inversion recovery (SPAIR)) with the following parameters: SENSE reduction factor, 3; averages, 2; TR/TE, 5336/61 ms; matrix, 240 × 240; FOV, 36 × 36 cm; in-plane voxel size, 1.5 mm; slice thickness, 5 mm; and gap, 0. Diffusion gradients were applied in six directions with *b* values of 0, 100, and 800 s/mm^2^. The total diffusion imaging acquisition time was 3 min and 28 s.

### Clinical MRI interpretation

Clinical interpretations for all MRI studies were performed prospectively by fellowship-trained radiologists specializing in breast imaging. Lesions were assessed using American College of Radiology (ACR) BI-RADS breast MRI lexicon [26], and lesion kinetic features were measured using computer-assisted diagnosis (CAD) software (CADstream v. 5.2.7, Merge Healthcare, Chicago, IL). For DCE kinetics, enhancement curve types of persistent, plateau, or washout were categorized for each voxel by evaluating the change in signal intensity from the initial (at 120 s) to the final (at 480 s) post-contrast scan, with washout defined as > 10% decrease in signal intensity, persistent > 10% increase, and plateau < 10% change. Recorded lesion characteristics included the number of lesions per patient, lesion type (focus, mass, non-mass enhancement [NME]), size, DCE kinetic pattern of worst curve type (defined as most suspicious, with washout > plateau > persistent [26]), final BI-RADS assessment and recommendation.

This information was entered into our clinical MRI database. Because the clinical evaluation of DCE-MRI images was performed prospectively, radiologists were blinded to the result of histopathology at the time of interpretation. DTI data was not reviewed by radiologists for BI-RADS assessment. Histopathology results from CNB and/or excision biopsy were later extracted from the clinical record for the purposes of this study.

### DTI post-processing

DTI analysis was performed offline by trained research scientists who were blinded to the lesion pathology outcomes. Diffusion tensor images were first spatially registered using a commercially available 3D affine transformation algorithm (Diffusion Registration tool, Philips Healthcare, Best, The Netherlands), with *b* = 0 s/mm^2^ images as reference, to minimize the artifacts due to motion and eddy current-based image distortion [27]. Voxel-based DTI parametric maps were then calculated and analyzed using in-house custom semi-automated software developed in ImageJ (National Institutes of Health, Bethesda, MD) based on standard methods [28]. With DTI, the MRI signal obtained with diffusion weighting is reduced in intensity proportional to the water mobility and is described by *S* = *S*_0_*e*^–*b*D^, where *S*_0_ is the signal intensity without diffusion weighting, *S* is the signal intensity with diffusion weighting, *D* is the diffusion tensor, and *b* is the applied diffusion sensitization [17]. A diffusion tensor matrix is derived for each image voxel and diagonalized to obtain the diffusion tensor eigenvalues *λ*_1_, *λ*_2_, and *λ*_3_ and scalar values describing the magnitude or rate of diffusion along each of the three principal axes (from largest to smallest) of the diffusion tensor ellipsoid (in mm^2^/s). From those, a number of other rotationally invariant DTI parameters can be calculated. ADC (also known as mean diffusivity, MD, or averaged diffusivity, *D*_av_), which describes the degree of mobility or hindrance of water molecules, is given by:

1 $$ \mathrm{ADC}=\mathrm{MD}=\frac{\lambda_1+{\lambda}_2+{\lambda}_3}{3}\kern0.75em \left({\mathrm{mm}}^2/\mathrm{s}\right) $$

(an alternative way to calculate ADC from the diffusion tensors vs. conventional DWI that does not account for anisotropy). Mean axial diffusivity is defined as *λ*_1_. Mean radial diffusivity is defined as the average of *λ*_2_ and *λ*_3_.

FA is a unitless measure of the degree of directionality of diffusion, ranging from 0 (completely isotropic) to 1 (completely anisotropic), given by:

2 $$ \mathrm{FA}=\frac{\sqrt{3}}{\sqrt{2}}\bullet \frac{\sqrt{{\left({\lambda}_1-\mathrm{ADC}\right)}^2+{\left({\lambda}_2-\mathrm{ADC}\right)}^2+{\left({\lambda}_3-\mathrm{ADC}\right)}^2}}{\sqrt{\lambda_1^2+{\lambda}_2^2+{\lambda}_3^2}}\kern1em $$

Additionally, the empirical parameter *λ*_1_ − *λ*_3_ is a non-normalized measure related to both diffusion mobility and directionality, which has been reported in previous studies to be significantly lower in malignant lesions compared to normal fibroglandular tissue and benign breast lesions [19, 22]. For qualitative interpretation, combined diffusion-weighted images were also calculated as the geometric average of unidirectional *b* = 800 s/mm^2^ images.

**Fig. 1:**
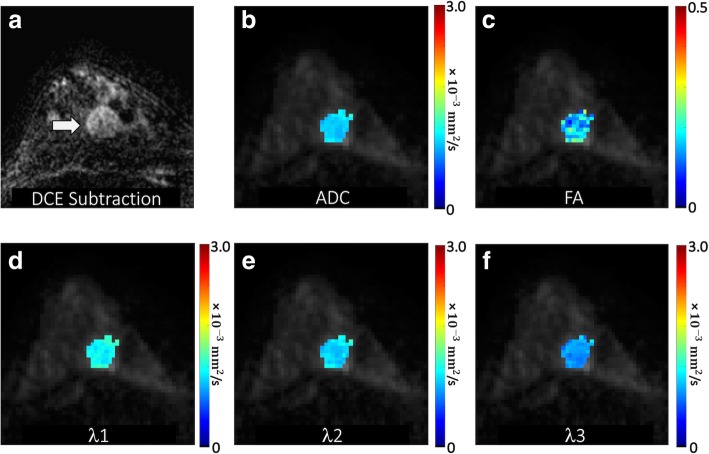
Malignant mass detected in a 47-year-old patient undergoing MRI to evaluate newly diagnosed cancer. **a** DCE post-contrast subtraction image demonstrates an additional 20-mm round mass with irregular margins in the posterior right breast 6 o’clock (arrow), assigned a BI-RADS category 4. DTI-derived parametric maps of **b** apparent diffusion coefficient (ADC), **c** fractional anisotropy, and eigenvalues **d** *λ*_1_, **e** *λ*_2_, and **f** *λ*_3_ are shown for the lesion regions overlaid in color on the *b* = 800 s/mm^2^ image. ADC, *λ*_1_, *λ*_2_, and *λ*_3_ are in units of 10^−3^ (mm^2^/s). The mass demonstrated low ADC (mean ADC = 1.09 × 10^−3^ mm^2^/s) with FA = 0.18, *λ*_1_ = 1.27 × 10^−3^ mm^2^/s, *λ*_2_ = 1.11 × 10^−3^ mm^2^/s, and *λ*_3_ = 0.87 × 10^−3^ mm^2^/s. Biopsy revealed a malignant grade 1 invasive ductal carcinoma

**Fig. 2:**
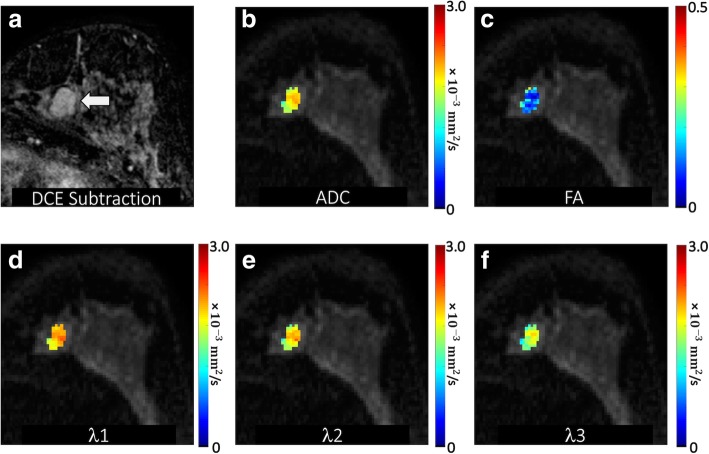
Benign mass detected in a 45-year-old patient undergoing MRI for high risk screening. **a** DCE post-contrast image demonstrates an 18-mm irregular mass with irregular margins in the left breast 7 o’clock (arrow), assigned a BI-RADS category 4. DTI-derived parametric maps of **b** apparent diffusion coefficient (ADC), **c** fractional anisotropy, and eigenvalues **d** *λ*_1_, **e** *λ*_2_, and **f** *λ*_3_ are shown for the lesion regions overlaid in color on the *b* = 800 s/mm^2^ image. ADC, *λ*_1_, *λ*_2_, and *λ*_3_ are in units of 10^−3^ (mm^2^/s). The mass demonstrated high ADC (mean ADC = 2.00 × 10^−3^ mm^2^/s) and very low FA (FA = 0.10), with *λ*_1_ = 2.20 × 10^−3^ mm^2^/s, *λ*_2_ = 2.00 × 10^−3^ mm^2^/s, and *λ*_3_ = 1.80 × 10^−3^ mm^2^/s. Ultrasound-guided biopsy revealed benign fibroadenoma

**Fig. 3:**
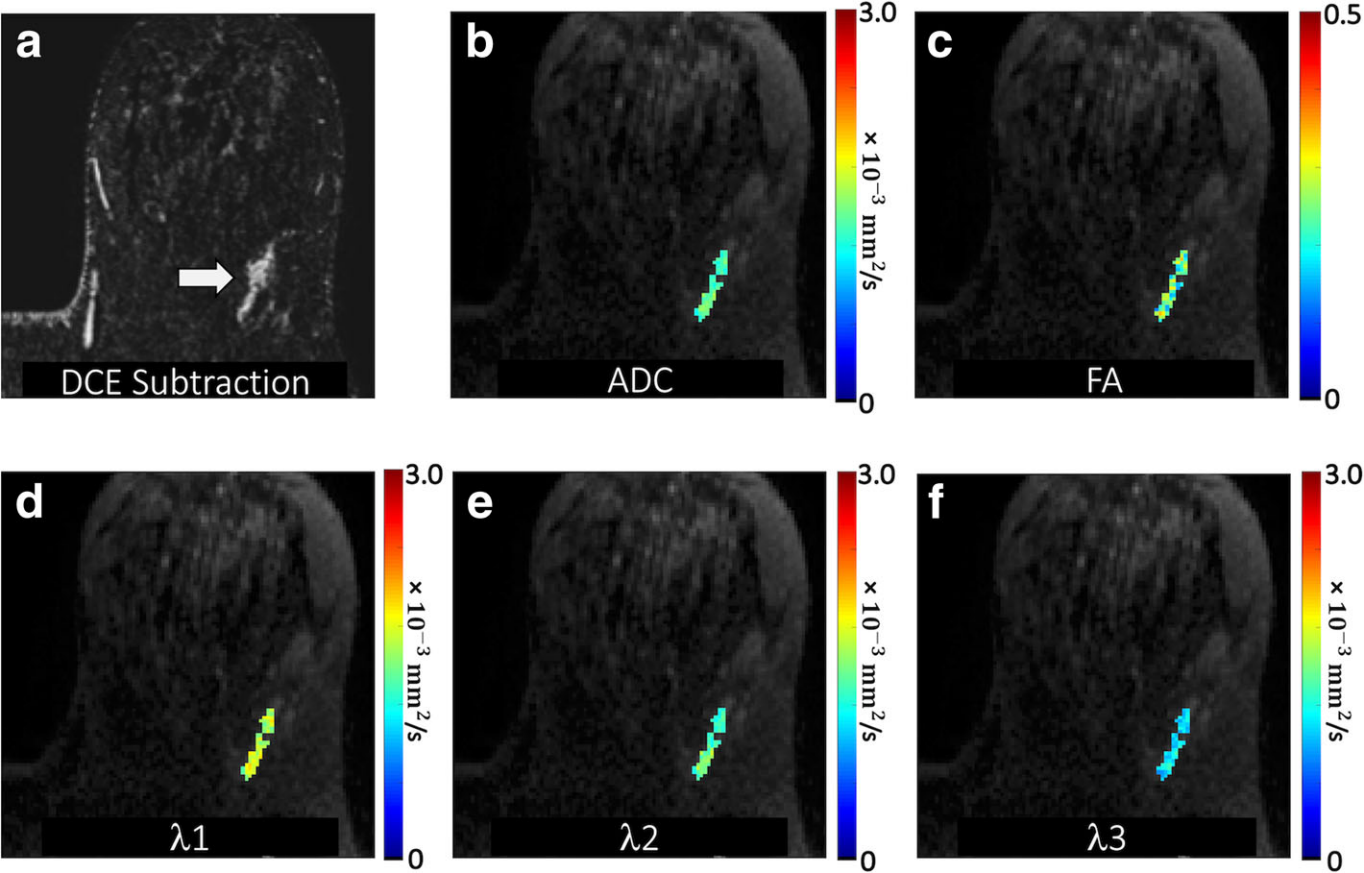
Malignant non-mass enhancement detected in a 58-year-old patient undergoing MRI for high risk screening. **a** DCE post-contrast image demonstrates a 33-mm linear heterogeneous non-mass enhancement in the posterior left breast 2 o’clock (arrow), assigned a BI-RADS category 4. DTI-derived parametric maps of **b** apparent diffusion coefficient (ADC), **c** fractional anisotropy, and eigenvalues **d** *λ*_1_, **e** *λ*_2_, and **f** *λ*_3_ are shown for the lesion regions overlaid in color on the *b* = 800 s/mm^2^ image. ADC, *λ*_1_, *λ*_2_, and *λ*_3_ are in units of 10^−3^ (mm^2^/s). The lesion demonstrated moderate ADC (mean ADC = 1.47 × 10^−3^ mm^2^/s) with FA = 0.25, *λ*_1_ = 1.83 × 10^−3^ mm^2^/s, *λ*_2_ = 1.47 × 10^−3^ mm^2^/s, and *λ*_3_ = 1.11 × 10^−3^ mm^2^/s. MR-guided biopsy revealed DCIS

**Fig. 4:**
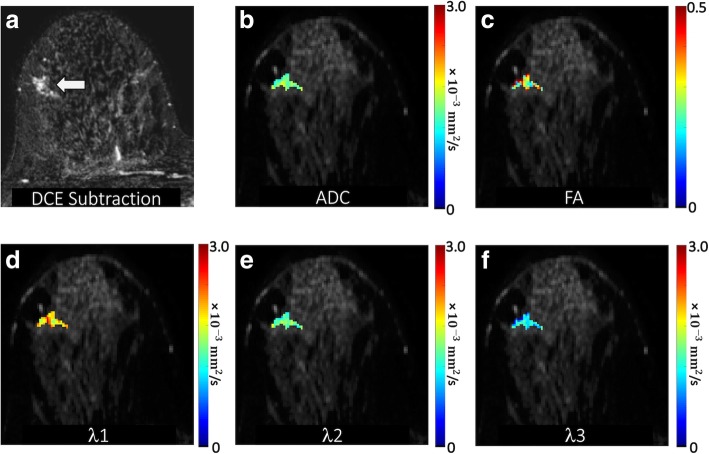
Benign non-mass enhancement detected in a 40-year-old patient undergoing MRI to evaluate newly diagnosed cancer. **a** DCE post-contrast image demonstrates a 20-mm focal heterogeneous non-mass enhancement in the middle right breast 9 o’clock (arrow), assigned a BI-RADS category 4. DTI-derived parametric maps of **b** apparent diffusion coefficient (ADC), **c** fractional anisotropy, and eigenvalues **d** *λ*_1_, (**e**) *λ*_2_, and (**f**) *λ*_3_ are shown for the lesion regions overlaid in color on the *b* = 800 s/mm^2^ image. ADC, *λ*_1_, *λ*_2_, and *λ*_3_ are in units of 10^−3^ (mm^2^/s). The lesion demonstrated moderate ADC (mean ADC = 1.56 × 10^−3^ mm^2^/s) and high FA (FA = 0.39), with *λ*_1_ = 2.13 × 10^−3^ mm^2^/s, *λ*_2_ = 1.58 × 10^−3^ mm^2^/s, and *λ*_3_ = 0.97 × 10^−3^ mm^2^/s. MR-guided biopsy revealed benign usual ductal hyperplasia

A region of interest (ROI) corresponding to the BI-RADS 4 or 5 lesion on DCE-MRI was manually defined on DTI under the supervision of an experienced radiologist. This ROI was selected on the combined DWI at the central slice of the lesion to include any hyperintensity and to avoid obvious areas of cyst, necrosis, or fat by referring to T1- and T2-weighted images. A semi-automated thresholding tool enabled exclusion from the ROI any voxels with very low DWI signal intensity corresponding to adipose or normal fibroglandular tissue [29]. The ROI was propagated to all DTI parametric maps, and the mean voxel value was calculated to characterize the lesion on each map. Propagating lesion ROIs directly from DCE-MRI was not possible due to the common spatial distortions inherent to echo-planar imaging-based DTI datasets (caused by B0 field inhomogeneities). Examples of breast DTI parametric maps are shown for several benign and malignant lesions in Figs. 1, 2, 3, and 4.

### Statistical analysis

An analysis was performed at the lesion level, and the primary outcome was histopathological determination of malignant vs. benign. Univariate associations between malignancy and clinical, DCE-MRI, and DTI parameters were explored using generalized estimating equations (GEE)-based logistic regression to account for any correlation between multiple lesions from the same woman. The area under the receiver operating characteristic (ROC) curve (AUC) and odds ratio (OR) were calculated for each parameter. Any variable which appeared to be right-skewed based on visual inspection of the histogram was log-transformed prior to inclusion in the model. ORs for continuous variables were scaled to correspond to the difference per 1-SD increase in the variable. Spearman’s rank correlation was used to examine the pairwise relationships between continuous variables.

Multivariate logistic regression models were developed using the least absolute shrinkage and selection operator (LASSO), which is a machine learning technique that simultaneously performs variable selection and parameter regularization to limit overfitting [30]. The LASSO penalty/regularization parameter was selected to minimize the model deviance estimated using leave-one-patient-out cross-validation. Three primary models were generated based on (1) Clinical/DCE-MRI parameters only, (2) DTI parameters only, and (3) the combination of both sets of parameters. Due to substantial collinearity among the DTI parameters, the primary DTI-based models were developed considering ADC and FA only. Sensitivity analyses to determine how model performance was impacted by changes in specific input variables were also performed where ADC was replaced with axial and radial diffusivity and where FA was replaced with *λ*_1_ − *λ*_3_. Model performance for discriminating between malignant and benign lesions was summarized using the AUC after optimism adjustment using the bootstrap to account for training and testing using the same dataset [31]. The bootstrap was also used to compare AUC estimates from different models. Bootstrap resampling was performed by patient rather than by lesion to account for the non-independence of lesions from the same patient.

An exploratory subgroup analysis of DTI parameters within the lesion groups defined by size (< 1 cm and ≥ 1 cm) and type (masses and non-masses) was conducted to assess whether the relationships between the DTI parameters and malignancy differed between any of these groups. This was done using GEE-based logistic regression models with interaction terms corresponding to the comparisons of interest, specifically size × ADC, type × ADC, size × FA, and type × FA. Lastly, a post-hoc multivariate LASSO model was developed, which included all Clinical/DCE-MRI parameters, DTI parameters, and the same interactions between size, type, and DTI parameters. All statistical calculations were performed using the statistical computing language R (version 3.1.1; R Foundation for Statistical Computing, Vienna, Austria). Throughout, two-sided tests were used, with statistical significance defined as *p* < 0.05.

## Results

### Patient cohort and lesion characteristics

Two hundred sixty-six women with 354 MRI-detected BI-RADS category 4 or 5 lesions were enrolled in our study prior to CNB and/or surgical excision. Of those, DTI was not performed in the MRI examinations for 23 women (24 lesions) and had technical or image post-processing issues in 13 women (16 lesions). Another 31 women (69 lesions) were then excluded: 42 lesions lacked adequate reference standard to determine the pathologic outcome, and 27 lesions were not evaluable because poor image quality or small lesion size precluded DTI measures. Lastly, 5 women with 7 lesions were excluded for incomplete clinical data: 2 women (2 lesions) in whom BPE was not evaluable due to prior mastectomy and 3 women (5 lesions) who did not have a recent mammogram available to assess breast density. After all exclusions, there were 238 lesions in 194 women included in the analysis.

Patient and lesion characteristics are summarized in Table 1. The median patient age was 51 years (range 23 to 83 years). The clinical indication for breast MRI was screening or problem solving in 68 (35.1%) patients and evaluating the extent of disease for newly diagnosed breast cancer in 128 (64.9%) patients. The majority (80.9%) of patients had a single BI-RADS 4 or 5 lesion detected on MRI while 15.5% had 2 lesions and 3.6% had 3 lesions. The median lesion diameter was 1.1 cm (range 0.4 to 11.4 cm), and most lesions were masses (*n* = 135, 56.7%), were classified as BI-RADS 4 (*n* = 218, 91.6%), and demonstrated washout on delayed phase DCE images (*n* = 198, 83.2%). Ninety-five of 238 (39.9%) lesions were malignant and 143 (60.1%) were benign. Lesion subtype information was available in 90 of 95 malignancies, which is summarized in Table 1.

**Table 1:** Patient and lesion characteristics

Patients (*N* = 194)	Value
Age, years	51 (23–83)
Menopausal status	
Pre	93 (47.9)
Post	101 (52.1)
Indication	
New cancer	126 (64.9)
Screening/problem solving	68 (35.1)
Breast density	
Fatty	5 (2.6)
Scattered fibroglandular	47 (24.2)
Heterogeneously dense	108 (55.7)
Dense	34 (17.5)
Background parenchymal enhancement	
Minimal	58 (29.9)
Mild	79 (40.7)
Moderate	37 (19.1)
Marked	20 (10.3)
Lesions per patient	
1 lesion	157 (80.9)
2 lesions	30 (15.5)
3 lesions	7 (3.6)
Lesions (*N* = 238)	
Largest diameter, cm	
< 1.0 cm	98 (41.2)
1.0–1.9 cm	71 (29.8)
2.0–3.9 cm	38 (16.0)
≥ 4.0 cm	31 (13.0)
Type	
Mass	135 (56.7)
NMLE	99 (41.6)
Focus	4 (1.7)
Delayed phase kinetics(most suspicious)	
Persistent	6 (2.5)
Plateau	34 (14.3)
Washout	198 (83.2)
BI-RADS	
4	218 (91.6)
5	20 (8.4)
Histopathology	
Malignant	95 (39.9)
Benign	143 (60.1)
Cancer subtype (*n* = 90*)	
Invasive	73 (81.1)
DCIS	17 (18.9)
Benign subtype (*n* = 143)	
Fibroadenoma	25 (17.5)
Fibrocystic changes	21 (14.7)
Fibrosis	14 (9.8)
Usual ductal hyperplasia	12 (8.4)
Apocrine metaplasia	11 (7.7)
Lobular neoplasia (LCIS, ALH)	9 (6.3)
Papilloma	9 (6.3)
Adenosis	8 (5.6)
Pseudoangiomatous stromal hyperplasia	6 (4.2)
Inflammation	5 (3.5)
Atypical ductal hyperplasia	4 (2.8)
Fibroadenomatoid change	4 (2.8)
Normal breast tissue	4 (2.8)
Other miscellaneous	11 (7.7)

Values are median (range) or no. (%)

*NME* non-mass enhancement, *DCIS* ductal carcinoma in situ, *LCIS* lobular carcinoma in situ, *ALH* atypical lobular hyperplasia

*Five malignancies did not have a cancer subtype available

### Performance of DCE-MRI and DTI parameters in discriminating malignant and benign lesions

In univariate analysis, all clinical and DCE-MRI parameters evaluated were significantly associated with malignancy except for mass vs. non-mass lesion type (*p* = 0.98), as summarized in Table 2. From DTI, lower ADC was significantly associated with malignancy (OR = 0.37 per 1-SD increase, *p* < 0.001), as were axial (OR = 0.42, *p* < 0.001) and radial (OR = 0.40, *p* < 0.001) diffusivity. Higher FA was significantly associated with malignancy (OR = 1.45, *p* = 0.007), though *λ*_1_ − *λ*_3_ was not (OR = 0.96, *p* = 0.77).

**Table 2:** Univariate analysis of patient and lesion characteristics (DCE+DTI) for discriminating between malignant and benign lesions

	Pathology status*				Univariate model		
	Malignant (*N* = 95)	Benign (*N* = 143)	AUC	(95% CI)	OR^†^	(95% CI)	*p* value
Clinical/DCE-MRI parameters							
Age, years	54.7 ± 11.3	49.3 ± 11.8	0.63	(0.56–0.71)	1.60	(1.19, 2.16)	0.002
Post-menopausal	61 (64.2)	67 (46.9)	0.59	(0.52–0.66)	2.04	(1.14, 3.62)	0.016
MRI indication: known cancer	77 (81.1)	84 (58.7)	0.61	(0.55–0.67)	3.00	(1.54, 5.85)	0.001
Dense breasts	57 (60.0)	117 (81.8)	0.61	(0.55–0.67)	0.33	(0.18, 0.62)	0.001
BPE category (1–4)	1.9 ± 1.0	2.3 ± 0.9	0.62	(0.54–0.70)	0.65	(0.47, 0.88)	0.006
Lesion size, cm^‡^	26.9 ± 27.1	16.3 ± 17.7	0.64	(0.57–0.71)	1.66	(1.27, 2.16)	< 0.001
Mass vs. NMLE/focus	54 (56.8)	81 (56.6)	0.50	(0.43–0.57)	1.01	(0.58, 1.76)	0.98
Washout on delayed phase kinetics	87 (91.6)	111 (77.6)	0.57	(0.53–0.61)	3.14	(1.41, 7.00)	0.005
BI-RADS 5 vs. 4	17 (17.9)	3 (2.1)	0.58	(0.53–0.62)	10.17	(2.11, 49.10)	0.004
DTI parameters							
Mean ADC, 10^−3^ mm^2^/s	1.26 ± 0.32	1.55 ± 0.30	0.75	(0.68–0.82)	0.37	(0.25, 0.54)	< 0.001
Mean axial diffusivity, 10^−3^ mm^2^/s	1.62 ± 0.41	1.91 ± 0.36	0.73	(0.66–0.80)	0.42	(0.28, 0.64)	< 0.001
Mean radial diffusivity, 10^−3^ mm^2^/s	1.08 ± 0.35	1.37 ± 0.33	0.74	(0.67–0.81)	0.40	(0.28, 0.59)	< 0.001
Mean FA^‡^	0.28 ± 0.15	0.23 ± 0.13	0.61	(0.53–0.68)	1.45	(1.11, 1.91)	0.007
Mean *λ*_1_ − *λ*_3_^‡^, 10^−3^ mm^2^/s	0.69 ± 0.46	0.69 ± 0.40	0.52	(0.44–0.60)	0.96	(0.74, 1.25)	0.77

*AUC* area under the ROC curve, *ROC* receiver operating characteristic curve, *OR* odds ratio for malignancy, *CI* confidence interval

*Values are no. (%) or mean ± SD

^†^For continuous variables, ORs are scaled to show change per 1-SD increase in the corresponding variable

^‡^Variable was log-transformed prior to inclusion in the logistic regression model to reduce right-skewness

NS = variable was included as a candidate predictor but was not selected by the LASSO; A blank cell indicates that the corresponding variable was not included as a candidate predictor in the model

There was substantial collinearity among the DTI parameters, at least in part due to their definitions which are all functions of the three diffusion eigenvalues. The pairwise correlations of ADC with axial (*r* = 0.81) and radial (*r* = 0.95) diffusivity were high, as was the correlation between FA and *λ*_1_ − *λ*_3_ (*r* = 0.89). FA was correlated with ADC (*r* = − 0.51), primarily through its correlation with radial diffusivity (*r* = − 0.73) rather than the axial component (*r* = 0.04). By contrast, *λ*_1_ − *λ*_3_ was only weakly correlated with ADC (*r* = − 0.07) because *λ*_1_ − *λ*_3_ was positively correlated with axial diffusivity (*r* = 0.48) but negatively correlated with radial diffusivity (*r* = − 0.36).

**Fig. 5:**
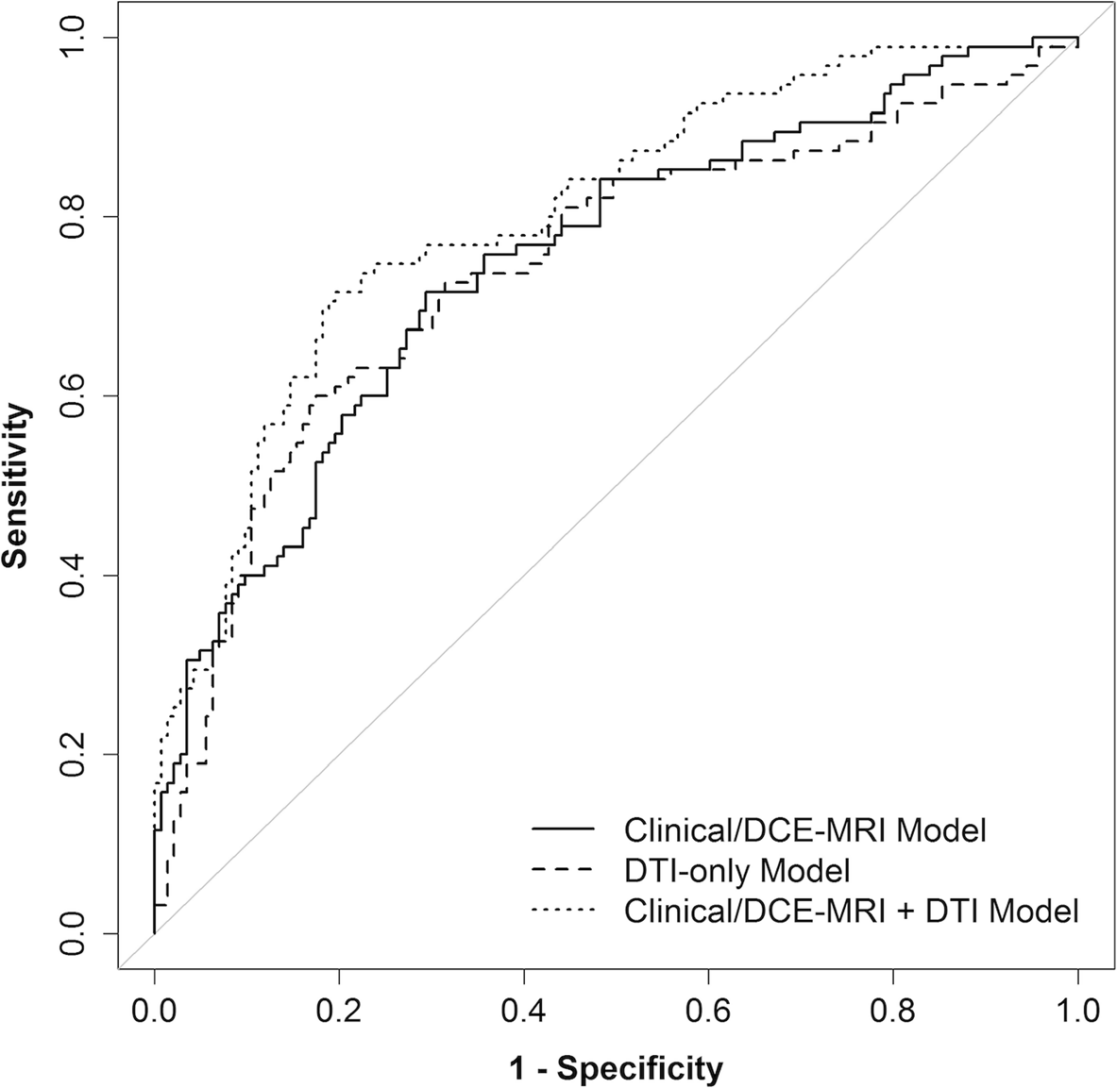
Cross-validated ROC curves for Clinical/DCE-MRI-only, DTI-only, and Clinical/DCE-MRI+DTI models to discriminate malignant and benign lesions. The bootstrap-adjusted AUC estimates were 0.76 (95% CI 0.71–0.83), 0.75 (95% CI 0.68–0.82), and 0.81 (95% CI 0.78–0.88), respectively. The Clinical/DCE-MRI+DTI model had a significantly higher AUC than the Clinical/DCE-MRI model (*p* < 0.001) and DTI-only model (*p* = 0.002)

The multivariate LASSO models are summarized in Table 3. The LASSO selected all of the Clinical/DCE-MRI parameters except post-menopausal status and selected only ADC (OR = 0.41 per 1-SD increase) for the DTI-only model. In the combined Clinical/DCE-MRI+DTI model, the LASSO selected both ADC (OR = 0.41) and FA (OR = 0.88). The Clinical/DCE-MRI model (AUC = 0.76) and DTI-only model (AUC = 0.75) had similar discrimination performance (ΔAUC = − 0.01, 95% CI − 0.10 to 0.06, *p* = 0.54). The Clinical/DCE-MRI+DTI model (AUC = 0.81) had significantly better performance than both the Clinical/DCE-MRI model (ΔAUC = 0.05, 95% CI 0.02 to 0.10, *p* < 0.001) and the DTI-only model (ΔAUC = 0.07, 95% CI 0.03 to 0.13, *p* = 0.002). Corresponding ROC curves are shown in Fig. 5. Since the OR for FA was relatively modest at 0.88, the incremental impact of FA was further examined. The performance of the Clinical/DCE-MRI+DTI model was compared with a Clinical/DCE-MRI+ADC model (including the same parameters but without FA), and the improvement in AUC from adding FA was not significant overall (ΔAUC = 0.00, 95% CI − 0.01 to 0.02, *p* = 0.81). The performance results from all of these models were very similar when the models were re-fit using axial and radial diffusivity instead of ADC and using *λ*_1_ − *λ*_3_ instead of FA.

**Table 3:** Multivariate LASSO models for discriminating between malignant and benign lesions

	Odds ratios*			
	Clinical/DCE-MRI	DTI only	Clinical/DCE-MRI+ADC	Clinical/DCE-MRI+DTI
Clinical/DCE-MRI parameters				
Age, per 1-SD increase	1.23		1.16	1.17
Post-menopausal	NS		NS	NS
MRI indication: known cancer	1.64		1.90	1.88
Dense breasts	0.45		0.64	0.61
BPE category, per 1-category increase	0.68		0.65	0.66
Lesion size^†^, per 1-SD increase	1.96		1.95	1.85
Mass vs. NMLE/focus	2.30		2.01	1.89
Washout on delayed phase kinetics	2.92		2.52	2.43
BI-RADS 5 vs. 4	4.47		2.56	2.36
DTI parameters				
Mean ADC, per 1-SD increase		0.41	0.44	0.41
Mean FA^†^, per 1-SD increase		NS		0.88
Bootstrap-adjusted AUC	0.76	0.75	0.81	0.81
(95% CI)	(0.71, 0.83)	(0.68, 0.82)	(0.77, 0.88)	(0.78, 0.88)

*For continuous variables, ORs are scaled to show change per 1-SD increase in the corresponding variable

^†^Variable was log-transformed prior to inclusion in the logistic regression model to reduce right-skewness

### Subgroup analysis of DTI parameters in small vs. large and mass vs. non-mass lesions

**Fig. 6:**
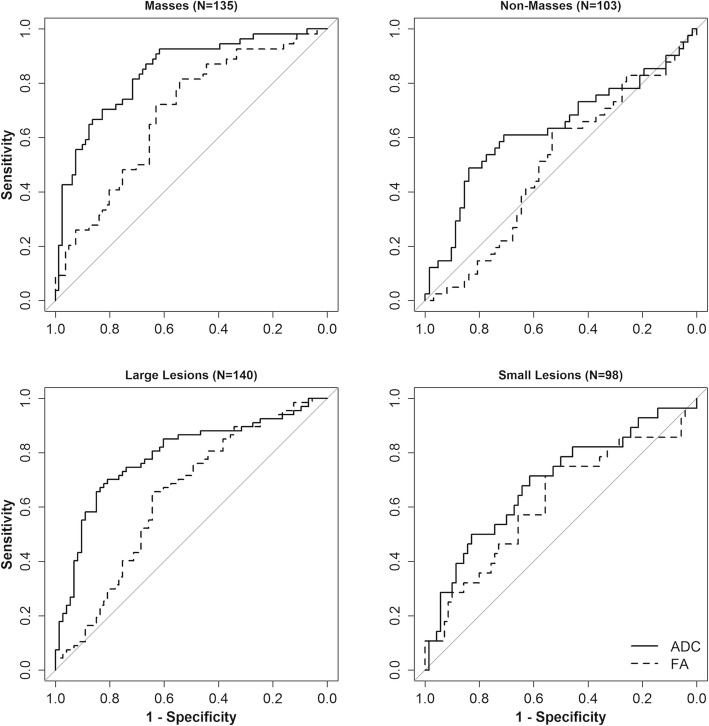
ROC curves for individual DTI parameters within the lesion subgroups defined by type or size. AUC estimates were significantly higher in masses than non-masses for both ADC (AUC 0.84 [95% CI 0.77–0.91] vs. 0.63 [95% CI 0.52–0.75], *p* = 0.002) and FA (AUC 0.69 [95% CI 0.59–0.79] vs. 0.50 [95% CI 0.40–0.61], *p* = 0.013). By contrast, AUC estimates were not significantly different between large (≥ 1 cm) and small (< 1 cm) lesions for both ADC (AUC 0.79 [95% CI 0.71–0.86] vs. 0.69 [95% CI 0.57–0.81], *p* = 0.18) and FA (AUC 0.64 [95% CI 0.55–0.73] vs. 0.62 [95% CI 0.49–0.75] *p* = 0.80)

An exploratory subgroup analysis was conducted of small (< 1 cm, *n* = 98) vs. large (≥ 1 cm, *n* = 140) lesions and mass (*n* = 135) vs. non-mass (NME *n* = 99; foci *n* = 4) lesions to assess how the DTI parameters were related to malignancy within these lesion subgroups. In univariate modeling, the predictive values of ADC and FA for malignancy were not significantly different between small and large lesions (*p* = 0.23 and *p* = 0.43, respectively) but were different between masses and non-masses (*p* = 0.002 and *p* = 0.006, respectively), Fig. 6. Specifically, ADC was more strongly predictive of malignancy in masses (OR = 0.19, *p* < 0.001) than non-masses (OR = 0.64, *p* = 0.060), while FA was significantly predictive only for masses (OR = 2.02, *p* < 0.001, Table 4) in univariate analysis.

**Table 4:** Exploratory modeling for the association of DTI parameters with malignancy in different lesion types

DTI variable	Pathology status				Univariate model			Multivariate model*		
	Malignant		Benign							
	No.	Mean ± SD	No.	Mean ± SD	OR	(95% CI)	*p* value	OR	(95% CI)	*p* value
ADC, 10^−3^ mm^2^/s										
Mass	54	1.19 ± 0.27	81	1.59 ± 0.30	0.19	(0.10–0.36)	< 0.001	0.23	(0.13–0.43)	< 0.001
Non-mass	41	1.35 ± 0.36	62	1.49 ± 0.30	0.64	(0.40–1.02)	0.060	0.42	(0.22–0.84)	0.013
					(*p* = 0.002^†^)			(*p* = 0.20^†^)		
FA										
Mass	54	0.31 ± 0.17	81	0.21 ± 0.12	2.02	(1.36–3.01)	< 0.001	1.62	(0.97–2.71)	0.067
Non-mass	41	0.24 ± 0.11	62	0.26 ± 0.14	0.94	(0.64–1.38)	0.74	0.54	(0.29–1.01)	0.053
					(*p* = 0.006^†^)			(*p* = 0.009^†^)		

*The multivariate model included ADC, FA, mass vs. non-mass type, log(lesion size), and interactions between mass/non-mass and ADC and FA; ORs were scaled to show change per 1-SD increase in the corresponding variable; FA was log-transformed prior to inclusion in the logistic regression model to reduce right-skewness

^†^Wald test comparing the ORs corresponding to masses and non-masses

Since both lesion type and size were related—non-masses (82/103; 80%) were more likely to be large (> 1 cm) than masses (58/135; 43%, *p* < 0.0001)—and ADC and FA were moderately correlated (r = − 0.51), they were included together in a multivariate model to assess their independent associations with malignancy in mass and non-mass groups (Table 4). The predictive value of FA remained significantly different between masses and non-masses in the multivariate model (OR 1.62 vs. 0.54, *p* = 0.009). Interestingly, the OR values implied opposite independent associations of FA with malignancy in the two groups, with malignant masses having higher FA values than benign masses but malignant non-masses having *lower* FA than benign non-masses. By contrast, the ORs for ADC were no longer significantly different between masses and non-masses in the multivariate model (OR 0.23 vs. 0.42, *p* = 0.20), with lower ADC values associated with malignancy in both lesion type groups.

We further explored why the FA association with malignancy varied between masses and non-masses. Of the malignant lesions, 51 of 54 (94.4%) masses were invasive cancers compared to 22 of 36 (61.1%) non-masses (*p* < 0.001). FA was significantly lower in DCIS lesions compared to invasive lesions (0.22 vs. 0.30, *p* = 0.026). On the other hand, FA was significantly higher in benign non-masses than in benign masses (0.26 vs. 0.21, *p* = 0.040). Among benign lesions, masses were most commonly fibroadenoma (25/83 [30.1%]), while non-masses were most commonly fibrocystic change (15/60 [25.0%]) and usual ductal hyperplasia (10/60 [16.7%]).

**Fig. 7:**
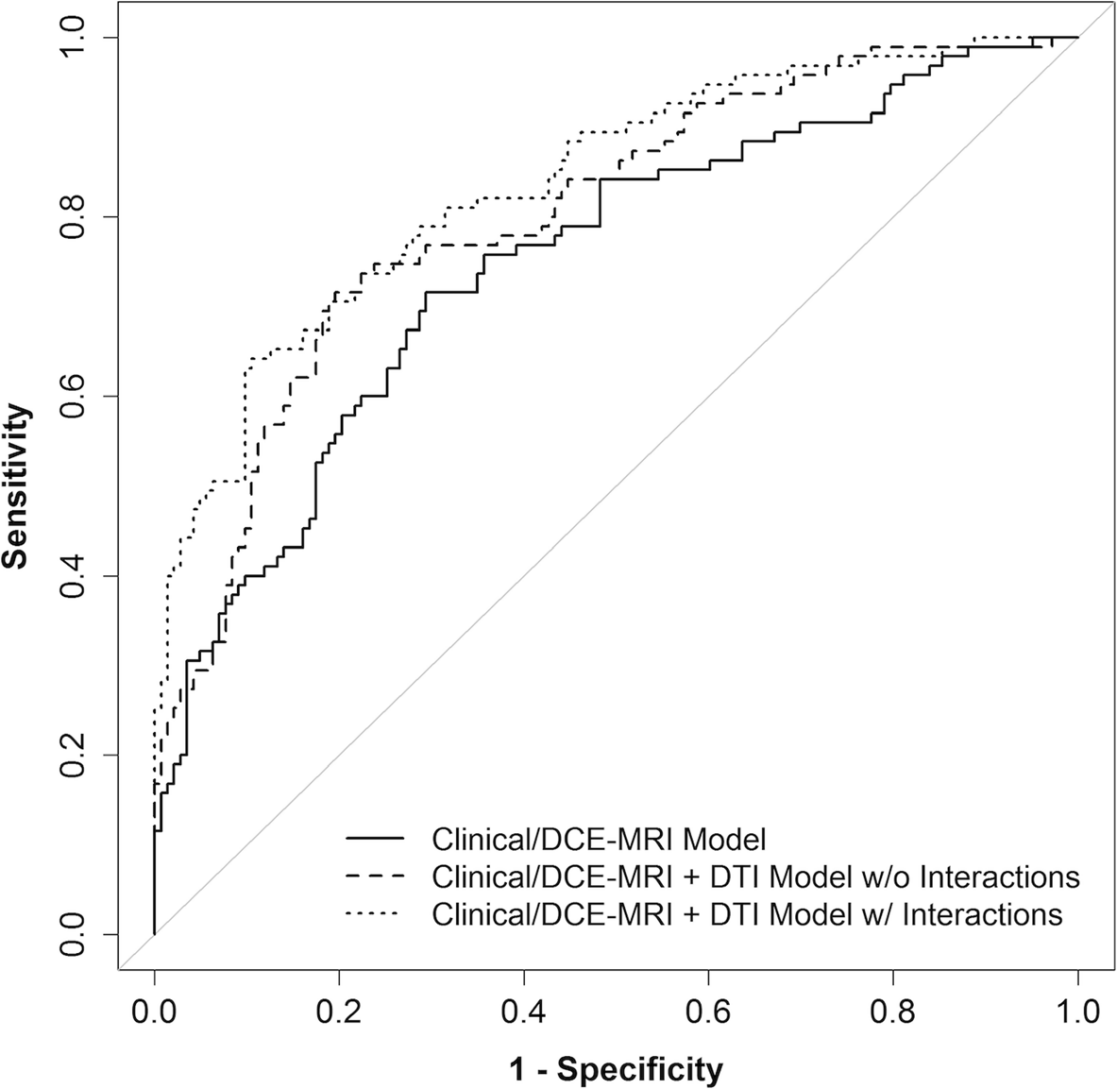
Comparison between the cross-validated ROC curve for Clinical/DCE-MRI+DTI+interactions model and curves for Clinical/DCE-MRI-only and Clinical/DCE-MRI+DTI models. The interaction terms were size × ADC, type × ADC, size × FA, and type × FA and allowed the model to have different odds ratios for ADC and FA for each size × type subgroup. The bootstrap-adjusted AUC estimates were 0.76 (95% CI 0.71–0.83), 0.81 (95% CI 0.78–0.88), and 0.85 (95% CI 0.82–0.90), respectively. The Clinical/DCE-MRI+DTI+interactions model had a significantly higher AUC than the Clinical/DCE-MRI+DTI model (*p* = 0.018)

To account for differential associations with malignancy depending on the lesion subgroup, an additional LASSO model combining Clinical/DCE-MRI parameters, DTI parameters, and size- and type-DTI interactions was then fit to assess the impact of incorporating the subgroup analysis results into the modeling (Table 5). This model allowed the ORs for ADC and FA to depend on whether the lesion was large or small and whether it was a mass or not. The resulting model with size- and type-DTI interactions (AUC = 0.85, 95% CI 0.82 to 0.90) demonstrated a substantial improvement in discrimination performance compared to the basic Clinical/DCE-MRI model (ΔAUC = 0.09, 95% CI 0.04 to 0.13, *p* < 0.001) and a modest though statistically significant improvement compared to the Clinical/DCE-MRI+DTI model without interactions shown in Table 3 (ΔAUC = 0.03, 95% CI 0.01 to 0.07, *p* = 0.018, Fig. 7).

**Table 5:** Multivariate Clinical/DCE-MRI+DTI LASSO model with type- and size-specific DTI parameters

	Odds ratios*	
	Model without interactions	Model with interactions^†^
Clinical/DCE-MRI parameters		
Age, per 1-SD increase	1.17	1.20
Post-menopausal	NS	NS
MRI indication: known cancer	1.88	1.84
Dense breasts	0.61	0.50
BPE category, per 1-category increase	0.66	0.59
Lesion size^‡^, per 1-SD increase	1.85	2.18
Mass vs. NMLE/focus	1.89	2.28
Washout on delayed phase kinetics	2.43	3.07
BI-RADS 5 vs. 4	2.36	2.97
DTI parameters		
Mean ADC, per 1-SD increase		
Small non-masses	0.41	1.00
Large non-masses	0.41	0.35
Small masses	0.41	0.36
Large masses	0.41	0.13
Mean FA^‡^, per 1-SD increase		
Small non-masses	0.88	0.47
Large non-masses	0.88	0.41
Small masses	0.88	1.17
Large masses	0.88	1.02
Bootstrap-adjusted AUC	0.81	0.85
(95% CI)	(0.78, 0.88)	(0.82, 0.90)

Small lesion, < 1 cm; large lesion, ≥ 1 cm

*For continuous variables, ORs are scaled to show change per 1-SD increase in the corresponding variable

^†^The model with interactions included addition terms corresponding to type × ADC, size × ADC, type × FA, and size × ADC, allowing all 4 subgroups (type × size) to have different ORs for ADC and different ORs for FA; the model without interactions contains 12 regression parameters (including the intercept), and the model with interactions contains 16 regression parameters

^‡^Variable was log-transformed prior to inclusion in the logistic regression model to reduce right-skewness

NS = variable was included as a candidate predictor but was not selected by the LASSO

## Discussion

The results of our prospective study of women with suspicious 3 T DCE-MRI-detected BI-RADS 4 and 5 lesions who underwent CNB and/or surgical excision showed that the addition of DTI to conventional breast MRI assessments may improve the ability to distinguish between benign and malignant lesions. Overall, malignancies exhibited lower ADC, lower axial (*λ*_1_) and radial ([*λ*_2_ + *λ*_3_]/2) diffusivity, and higher FA on DTI than benign lesions. Further analysis demonstrated that FA association with malignancy differed for masses and non-masses, and among the diffusion parameters, ADC contributed most significantly to the overall diagnostic performance. Machine learning-based LASSO modeling incorporating multiple clinical, conventional DCE and DTI parameters achieved high diagnostic performance in differentiating malignant from benign lesions (AUC = 0.85).

Prior studies have demonstrated that lesion size and worst delayed phase kinetics on DCE-MRI are significant predictors of malignancy [32–34], and the results of our study further validate the significance of these parameters, with malignant lesions being larger and more likely to demonstrate washout on delayed phase in both univariate and multivariate analyses. As expected, BI-RADS category 5 lesions were more likely to be malignant than BI-RADS 4 lesions. Lesion type (mass vs. non-mass) was not found to be a significant parameter. Other patient-level characteristics associated with higher odds for lesion malignancy were older age, post-menopausal status, lower BPE, and lower breast density. These findings agree with established evidence of increasing risk of breast cancer with age [29], as well as reduced mammographic breast density and BPE that go along with increasing age [35]. Furthermore, higher BPE (moderate or marked levels) has been shown to be associated with higher abnormal interpretation (BI-RADS 0,3,4,5) and biopsy rates and lower specificity (i.e., higher false-positive rate) [36].

The latest (fifth) edition of the ACR BI-RADS lexicon contains descriptors for lesion morphology and contrast kinetics but not for diffusion characteristics [26], which reflects the current clinical practice of DWI being used only in select imaging centers as an adjunctive technique to conventional DCE-MRI. Results from multiple single-center studies, including prior work at our institution, have demonstrated the value of quantitative ADC measures in discriminating malignant from benign lesions [15, 37]. Results of a recent multicenter trial further confirmed that by implementing an ADC cutoff, DWI has potential to reduce the rate of unnecessary biopsies prompted by conventional DCE-MRI [16]. The pathologic basis for impeded diffusion and lower ADC values in malignant lesions has been proposed to arise in part from the higher cellularity and more restricted extracellular environment of breast cancers compared to benign lesions, supported by imaging pathologic comparisons in some studies [11, 23]. In our study, univariate analysis identified ADC as a significant parameter, and LASSO modeling selected ADC for both the DTI-only and DCE+DTI models. Our findings add to a growing body of evidence supporting the use of ADC as an independent biomarker for differentiating between malignant and benign lesions.

In addition to the magnitude of diffusion provided by ADC, other DTI parameters provide information on the direction and anisotropy of diffusion, potentially allowing for further characterization of the underlying breast tissue. Although there is a general consensus in the literature supporting impeded diffusion (reflected by low ADC) as a feature of breast malignancies, there are conflicting results regarding the added utility of other DTI parameters in differentiating between malignant and benign lesions. FA is the most studied DTI parameter aside from ADC, and while some studies have reported higher FA in malignant lesions compared with benign ones [20, 23–25], others have found no significant difference [18, 21, 22]. The univariate analysis in our study showed mean FA to be significantly higher in malignant lesions compared to benign lesions. Mean axial (*λ*_1_) and radial ([*λ*_2_ + *λ*_3_]/2) diffusivity were also significantly lower in malignancies. On the other hand, the empirical parameter *λ*_1_ − *λ*_3_, a proposed alternate measure of diffusion directionality that has been reported to be lower in malignant lesions compared to normal breast tissue and benign lesions [19, 22], did not significantly distinguish malignant and benign lesions in our study.

Post-hoc subanalyses demonstrated interesting differences in FA association with malignancy based on the lesion type of mass or non-mass. Higher FA was associated with malignancy for masses, while lower FA was associated with malignancy for non-mass lesions in multivariate modeling, which has not been previously reported. Our findings suggest this opposite association of FA with malignancy likely relates to biologic differences of the typical benign pathologies represented within each lesion type. In our study, benign masses demonstrated lower FA than benign non-masses. Within masses, the most common false positives on MRI are fibroadenomas, where FA has been reported to be lower than for malignancies in multiple prior studies in addition to ours [20, 23–25] and attributed to their characteristic myxoid extracellular matrix and fibrous stroma with absent or compressed tubular structures [20]. On the other hand, within non-masses, common benign pathologies such as ductal hyperplasia or pseudoangiomatous stromal hyperplasia (PASH) may grow diffusely while maintaining some of the normal ductal architecture and native FA levels of the intervening fibroglandular tissue. Further investigation with more detailed pathological assessment is needed to better understand the microstructural and microenvironmental characteristics of breast lesions influencing diffusion anisotropy measures and how this information may be used to improve diagnostic accuracy. Regardless, our findings suggest DTI anisotropy metrics must be considered in the context with lesion type for diagnostic purposes.

The results of our multivariate LASSO analysis showed that a model incorporating all clinical factors (except menopausal status), DCE-MRI parameters, and DTI parameters (ADC and FA) and accounting for interactions achieved the best diagnostic performance in differentiating malignant from benign lesions, represented the highest AUC (0.85). Comparison of several multivariate models further demonstrated that among the DTI parameters, ADC contributed most significantly to the overall diagnostic performance and suggested FA added incremental value only after accounting for the interactions with lesion type. ADC maps can be acquired using the standard DWI technique with three orthogonal diffusion-sensitizing gradients, while multiple additional gradient directions must be applied to acquire sufficient data to reconstruct diffusion tensors in DTI. Our results suggest that using only the less time-consuming standard DWI acquisition in conjunction with conventional DCE-MRI may be sufficient for improving the diagnostic performance.

Along with demonstrating potential clinical utility, the study also identified that further technical developments are needed to address breast DTI image quality issues. Lesion evaluability on DTI was limited in part by technical issues inherent to the single-shot echo-planar imaging (EPI) technique. EPI is widely used for diffusion imaging but suffers from limited spatial resolution, spatial distortion, and frequent artifacts [38]. These issues are further magnified for breast imaging due to the particular challenges of off-isocenter imaging, air-tissue interfaces, and significant fat content in the breast [14]. To mitigate these potential sources of error, exams found on visual assessment to have significant imaging artifacts were excluded from the study, but these issues may still have contributed to reduced lesion evaluability in the remaining exams, with 27/272 (10%) lesions with adequate DTI quality and reference standard still deemed non-evaluable. The results of recent multicenter breast DWI trials also identified reliable image quality to be a challenge, with 13 to 29% of cases being excluded for DWI technical issues [16, 39, 40]. However, a range of emerging technical advancements in DWI acquisition strategies hold potential to improve image quality [41–44].

There are several strengths of our study. To our knowledge, our study contains the largest prospective cohort of patients to date who have undergone breast DTI for detecting breast malignancy. After excluding lesions without direct pathologic sampling and with other missing clinical data, 238 lesions included in our study were available for statistical analysis. Our study featured a prospective design, and interpreting radiologists and research associates conducting quantitative DTI analyses were blinded to the histopathologic results. All breast MRIs in our study were performed at high-field strength (3 T), which has been shown in prior studies to have improved diagnostic performance compared to 1.5 T due to superior signal-to-noise ratio (SNR), which can in turn enable increased spatial resolution [45–47]. To increase the robustness of our statistical analysis, we used an advanced machine learning-based LASSO modeling technique, which simultaneously performs variable selection and parameter regularization to limit overfitting, along with bootstrap optimism adjustment to account for training and testing using the same dataset.

There are also several important limitations to our study. First, although our study contains the largest cohort of breast DTI patients to date, it was performed at a single institution, which may limit the generalizability of our results. DTI was performed with minimal six gradient directions and with 1.5 × 1.5 × 5 mm spatial resolution. It is possible additional directions and/or higher spatial resolution could better elucidate directionality in breast lesions; however, this would require additional acquisition time and may incur additional motion and other artifacts. In order to minimize disruption of the clinical portion of breast MRI examinations and in case patients were not able to tolerate the full examination, DTI was performed after the completion of the standard MRI protocol including DCE-MRI (approximately 10 min after contrast injection). Although we have previously shown no significant effect on breast tumor ADC measures using our imaging protocol [48], which was also verified across multiple independent breast DWI studies [49], it may be preferable to acquire DTI before contrast injection to avoid any possible confounding effects on other DTI parameters. Furthermore, ROIs were manually defined on DTI maps for each exam after comparing DCE-MRI and DTI. Propagating lesion ROIs directly from DCE images was not reliable due to the spatial distortions common to EPI-based breast DWI and DTI datasets (caused by field inhomogeneities). Manual ROI definition is prone to operator dependence and sampling error, especially for irregularly shaped masses or NME. To address this limitation, we used a semi-automated ROI tool to avoid non-tumor voxels, which has been shown in a previous study to improve the inter-reader reproducibility of breast lesion ADC values without introducing bias vs. manual ROI measures [29]. Alternate ROI approaches such as sampling a small hotspot region could provide different performance results for distinguishing benign from malignant lesions.

## Conclusion

Evaluating a combination of clinical, DCE-MRI, and diffusion parameters may improve the ability to distinguish between benign and malignant lesions on breast MRI, thereby decreasing false-positive diagnoses and avoiding unnecessary biopsies. ADC was the most important diffusion parameter for distinguishing benign and malignant breast lesions, supporting continued use of standard DWI sequences for feasible clinical implementation. However, our results suggest DTI may enable further biologic characterization relating to variations in tumor microstructure and microenvironment, which warrants further investigation.

## Data Availability

The datasets generated and analyzed during the current study are not publicly available due to identifiable patient information but are available from the corresponding author on reasonable request.

## Abbreviations

DCE: Dynamic contrast enhanced
DWI: Diffusion-weighted imaging
ADC: Apparent diffusion coefficient
DTI: Diffusion tensor imaging
FA: Fractional anisotropy
HIPAA: Health Insurance Portability and Accountability Act
BI-RADS: Breast Imaging Reporting and Data System
CNB: Core needle biopsy
TR: Repetition time
TE: Echo time
FOV: Field of view
EPI: Echo-planar imaging
ACR: American College of Radiology
CAD: Computer-assisted diagnosis
NME: Non-mass enhancement
MD: Mean diffusivity
ROI: Region of interest
GEE: Generalized estimating equations
ROC: Receiver operating characteristic
AUC: Area under the receiver operating characteristic curve
OR: Odds ratio
LASSO: Least absolute shrinkage and selection operator
